# Successful Diagnosis of Neuroleptic Malignant Syndrome in an Unconscious Patient Using Amplitude-Integrated Electroencephalography: A Case Report

**DOI:** 10.7759/cureus.61927

**Published:** 2024-06-07

**Authors:** Shunsuke Nakamura, Atsuyoshi Iida, Kohei Tsukahara, Hiromichi Naito

**Affiliations:** 1 Emergency, Critical Care, and Disaster Medicine, Okayama University Graduate School of Medicine, Dentistry, and Pharmaceutical Sciences, Okayama, JPN; 2 Advanced Emergency and Critical Care Center, Okayama University Hospital, Okayama, JPN; 3 Advanced Emergency and Critical Care Center, Okayama University Hospital, okayama, JPN

**Keywords:** neuroleptic malignant syndrome (nms), seizure, muscle rigidity, dantrolene, convulsion, amplitude-integrated electroencephalography (aeeg)

## Abstract

Neuroleptic malignant syndrome (NMS) is a rare but life-threatening medical condition often characterized by altered consciousness and clinical features resembling seizures. This case report presents a unique and successful diagnosis of NMS in an unconscious patient with an unknown medical history. We demonstrate the potential utility of amplitude-integrated electroencephalography (aEEG) as a valuable tool for the differential diagnosis of seizure-like medical conditions, including NMS. The application of aEEG allowed for early diagnosis and prompt initiation of appropriate treatment, potentially contributing to improved patient outcomes.

## Introduction

The examination of patients with tremors and altered consciousness presents the challenge of distinguishing conditions requiring early intervention. Particularly, differential diagnosis for status epilepticus, neuroleptic malignant syndrome (NMS), and other medical diagnoses is important. When a patient lacks a detailed medical history on emergency admission, differential diagnosis is often difficult; therefore, thorough physical examinations and electroencephalography (EEG) are necessary for the treatment of seizures. The EEG conducted in the intensive care unit (ICU) serves not only for the detection of status epilepticus and recurrent subclinical seizures but also proves valuable for assessing disorders of consciousness and prognostication. However, facilities are lacking, as are experienced electroencephalographers capable of interpreting the EEG [1]. Amplitude-integrated electroencephalography (aEEG) facilitates ascertaining the presence of epileptic seizures by presenting amplitude summation over time, offering a means to decipher intricate encephalography waveforms that might challenge non-specialists. Hence, it allows emergency physicians with limited interpretation experience to interpret the report, as opposed to conventional EEG. We report a successful diagnosis of NMS where the patient was unconscious with tremors and without any information about his medical history. This report implies the potential use of aEEG for differential diagnosis of seizure-like medical conditions. Amplitude-integrated electroencephalography may contribute to the early diagnosis and treatment of seizure-like medical conditions, including NMS.

## Case presentation

A 49-year-old man was found unconscious on the roadside and brought to the emergency department. Emergency medical services found a generalized tonic convulsion at the scene. Upon arrival at our hospital, the patient was unconscious. In the absence of a medical history, his vital signs were as follows: a heart rate of 140 beats per minute in sinus rhythm, unmeasurable low blood pressure, a respiratory rate of 60 breaths per minute, and oxygen saturation of 99% (administered at 10 L/min with a reservoir mask). The Glasgow Coma Scale score was seven (E1, V1, M5), both pupil diameters were 3.5 mm, and light reflexes were sluggish. A body temperature of 41.2°C and a generalized tonic convulsion were observed. The patient's condition was stabilized with artificial respiration, fluid resuscitation, and catecholamine administration. The generalized tonic convulsion was controlled with 10 mg of diazepam 40 minutes after the emergency call. No abnormal findings were confirmed on head computed tomography. Blood analysis indicated a creatine kinase (CK) level of 646 IU/L, along with hyperammonemia (319 μg/dl), suggesting seizure activities. Laboratory analysis results are presented in Table 1.

**Table 1:** The patient's laboratory data on admission

Test	Result	Units	Reference values
White blood cell	18100	/μL	3300 - 8600
Red blood cell	5.01*10^6^	/μL	4.35 - 5.55*10^6^
Hemoglobin	16.0	g/dL	13.7 - 16.8
Hematocrit	46.2	%	40.7 - 50.1
Platelet	455*10^3^	/μL	155 - 348*10^3^
Aspartate aminotransferase	45	U/L	13 - 30
Alanine aminotransferase	31	U/L	10 - 42
Lactate dehydrogenase	390	U/L	124 - 222
Creatine kinase	646	U/L	59 - 248
Urea nitrogen	23.5	U/L	8 - 20
Creatinine	2.58	mg/dL	0.65 - 1.07
Total bilirubin	1.73	mg/dL	0.4 - 1.5
C-reactive protein	0.07	mg/dL	< 0.14
Sodium	141	mmol/L	138 - 145
Potassium	5.9	mmol/L	3.6 - 4.8
Chlorine	102	mmol/L	101 - 108
Calcium	10.4	mg/dl	8.8 - 10.1
Albumin	5.3	g/dL	4.1 - 5.1
Ammonia	319		40 - 80
Procalcitonin	0.189	ng/ml	< 0.05
Prothrombin time (PT)	11.1	second	11 - 13
Presepsin	231	pg/mL	< 314
PT(%)	87	%	80 - 120
PT-international normalized ratio	1.07		0.9 - 1.1
Activated partial thromboplastin time	22.5	second	25 - 40
Fibrinogen	342	mg/dL	200 - 400
D-dimer	2.9	μg/mL	< 1.0
Free triiodothyronine	3.21	pg/ml	2.30 - 4.00
Free thyroxine	1.93	ng/dl	0.97 - 1.69
Thyroid-stimulating hormone	0.94	μU/mL	0.33 - 4.05

**Figure 1:**
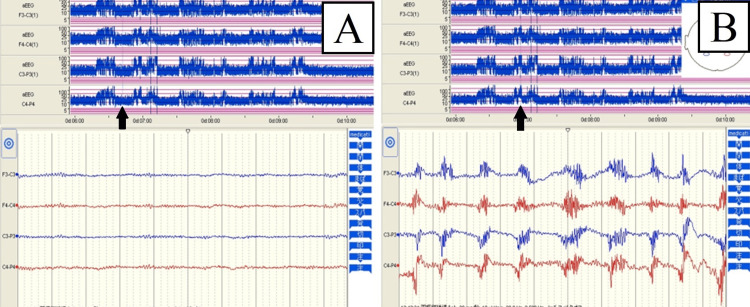
The aEEG waves on admission to the ICU (A) At rest (arrow), only normal rhythmic activity waves are present; (B) During convulsive-like movements (arrow), artifacts due to muscle rhythm are mixed in. aEEG: amplitude-integrated electroencephalography; ICU: intensive care unit

Urine drug tests showed negative results, and cerebrospinal fluid analysis showed normal pressure, clear fluid, and a normal cell count. The thyroid hormone levels were as follows: free triiodothyronine (fT3), 3.21 pg/ml; free thyroxine (fT4), 1.93 ng/dl; and thyroid-stimulating hormone (TSH), 0.94 mIU/L. There were no abnormal values suggestive of a crisis and no protruding eyeballs. The extrapyramidal symptoms of dystonia, dyskinesia, and slow movement were not seen at any time during hospitalization. The inflammatory markers C-reactive protein (CRP) and procalcitonin were only substandard or mildly elevated, and presepsin, which more acutely reflects infection, was substandard. After a little over an hour had elapsed since the hospital admission, generalized rigidity recurred. Given the patient's complete lack of background information at this point, prominent diagnostic considerations included status epilepticus and potential NMS. Upon admission to the ICU, aEEG was implemented, and that showed normal background activity during rest. Subsequent observation of aEEG brain waves during seizure-like movements revealed artifact interference due to muscular activity, with no evidence of epileptic waves. Because only the electromotive waves manifested during the seizure, movement was attributed to muscle rigidity (Figure 1). 

**Figure 2:**
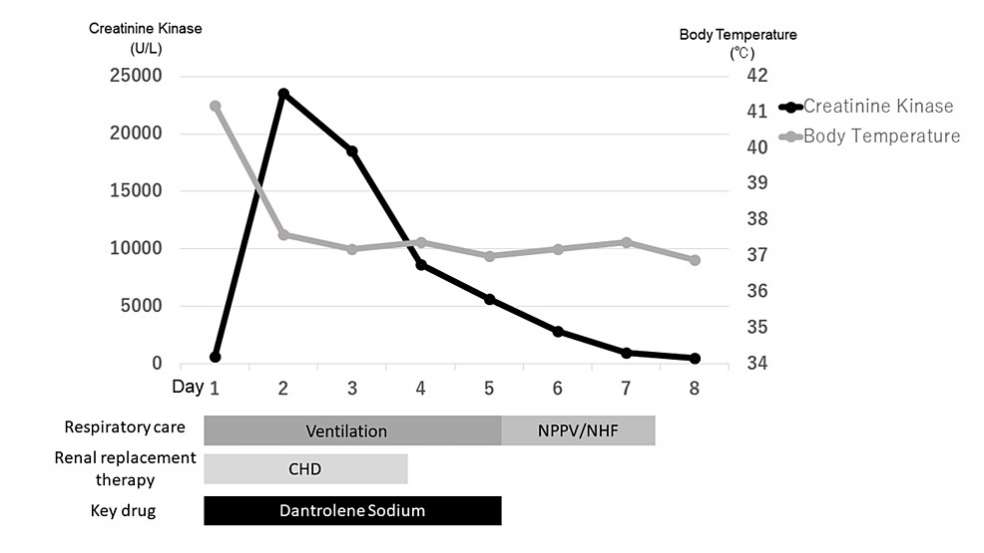
The patient's clinical course after admission Levels of CK peaked the day after admission, and dantrolene sodium administration was given every 12 hours until day five. CK: creatine kinase; NPPV: noninvasive positive-pressure ventilation; NHF: nasal high flow; CHD: continuous hemodialysis

Ultimately, profuse sweating, tremors, altered consciousness, tachycardia, blood pressure instability, leukocytosis, and elevated CK levels led to the diagnosis of NMS. A bolus of 40 mg, followed by 20 mg of dantrolene sodium every 12 hours, was administered. Due to hemodynamic instability and renal impairment, catecholamines and continuous renal replacement therapy were needed. The patient's condition gradually improved with successful extubation and dantrolene sodium administration was stopped on the fifth day. The clinical course of the patient is shown in Figure 2.

On the sixth day, his statement revealed that he had a history of psychiatric hospital visits and was taking biperiden, blonanserin, quetiapine, and levomepromazine, leading to a confirmed NMS diagnosis. The patient had a history of methamphetamine psychosis. There was no information that the patient regularly drank large amounts of alcohol, nor had he abstained from alcohol prior to admission. There were no serotonin agonists among the regular medications identified after admission. The patient was treated with quetiapine fumarate, nitrazepam, blonanserin, and lorazepam at appropriate doses after improvement. No side effects were observed throughout the hospitalization. He was transferred to a psychiatric hospital on the tenth day and has shown favorable post-discharge progress.

## Discussion

This case highlights the critical importance of early and accurate diagnosis in patients with altered consciousness resembling seizures. The use of aEEG enabled differentiating NMS from primary seizure disorders in an unconscious patient with an unknown medical history. Recognizing the absence of epileptic activity on the aEEG and correlating it with clinical findings led to timely intervention and ultimately a favorable outcome for the patient. Recently, aEEG has enabled convenient, rapid, and continuous brain wave monitoring in the ICU. The aEEG is characterized by its ability to visually distinguish between the sawtooth wave trend pattern characteristic of convulsive seizures and the high amplitude trend pattern caused by electromyogram (EMG) artifacts by displaying the amplitude of the EEG waveforms over time. On the other hand, the low number of electrode inductions results in reduced sensitivity, and artifacts also occur. Utilized for central nervous system monitoring in adult post-resuscitation care [2], aEEG is valuable for the early diagnosis of nonconvulsive status epilepticus [3]. Furthermore, in pediatric neuro-intensive care, aEEG is often used to detect seizures in neonates with hypoxic brain injury [4]. While imaging and cerebrospinal fluid tests are common in investigating altered consciousness, EEG monitoring provides compelling evidence for epilepsy. Hence, EEG measurement in patients with altered consciousness accompanied by tremors or muscle rigidity is clinically important. The application of aEEG facilitates real-time assessment of brain wave activity during tremor episodes at the point of care, serving as a robust diagnostic aid.

Neuroleptic malignant syndrome, a rare but potentially fatal drug-induced hyperthermia, presents with altered mental status, muscle rigidity, and autonomic dysfunction [5]. An international consensus proposed NMS diagnostic criteria in 2011 [6], but no definitive diagnostic criteria for NMS diagnosis have been established. Early NMS diagnosis and management are critical to prevent life-threatening complications such as rhabdomyolysis, acute respiratory failure, acute renal impairment, sepsis, and other systemic infections [7]. An EEG is sometimes applied to NMS cases. Caroff et al. reported in their review that EEG was performed in more than half of NMS cases, and non-focal, generalized slow waves have been found [6,8,9]. Rosebush reported a series of 24 NMS cases, and the EEG showed diffuse slow waves without focal abnormality in seven of the eight cases that were studied [10]. In our case, only background activity waves were observed at rest, with artifacts caused by muscle rhythm during convulsive-like movements. Dantrolene sodium, a muscle relaxant, is the first-line drug indicated for the pharmacologic treatment of NMS. The dopamine agonist bromocriptine may also be used. Short-term use of anxiolytics may be effective when psychiatric symptoms are prominent. Anxiolytics can also help reduce the symptoms of malignant syndromes because of their muscle relaxant effects. In cases of psychiatric exacerbations, electroconvulsive therapy may be effective for both the malignant syndrome and psychiatric symptoms. After symptom improvement, antipsychotics should be restarted at a low dose and continued or increased cautiously with or without relapse.

## Conclusions

Amplitude-integrated electroencephalography can serve as a valuable diagnostic tool for the early differentiation of seizure-like medical conditions, including NMS. In cases of unconscious patients with unclear medical histories, aEEG can aid in the rapid identification of non-epileptic etiologies, facilitating prompt and appropriate treatment. This report underscores the potential of aEEG in improving outcomes for patients with conditions that mimic seizures, emphasizing the need for its integration into clinical practice for such scenarios.
